# Bacteria-targeted imaging using vancomycin-based positron emission tomography tracers can distinguish infection from sterile inflammation

**DOI:** 10.1007/s00259-024-06997-z

**Published:** 2024-11-29

**Authors:** G. B. Spoelstra, L. M. Braams, F. F. A. IJpma, M. van Oosten, B. L. Feringa, W. Szymanski, P. H. Elsinga, Jan Maarten van Dijl

**Affiliations:** 1 Department of Nuclear Medicine and Molecular Imaging, University of Groningen, University Medical Center Groningen, Hanzeplein 1, Groningen, 9713GZ The Netherlands; 2 Department of Medical Microbiology and Infection Prevention, University of Groningen, University Medical Center Groningen, Hanzeplein 1, Groningen, 9713GZ The Netherlands; 3 Department of Trauma Surgery, University of Groningen, University Medical Center Groningen, Hanzeplein 1, Groningen, 9713GZ The Netherlands; 4 Stratingh Institute for Chemistry, University of Groningen, Nijenborgh 4, Groningen, 9747AG The Netherlands; 5 Department of Radiology, University of Groningen, University Medical Center Groningen, Hanzeplein 1, Groningen, 9713GZ The Netherlands; 6 Department of Medicinal Chemistry, Photopharmacology and Imaging, University of Groningen, Groningen Research Institute of Pharmacy, Antonius Deusinglaan 1, Groningen, 9713AV The Netherlands

**Keywords:** Bacterial infection imaging, PET, Antibiotic-based tracer, ^18^F-vancomycin, Vancomycin, In vivo, Myositis

## Abstract

**Introduction:**

Bacterial infections pose major challenges in medicine. To guide effective infection treatment, faster and more accurate diagnostic modalities are needed. Bacteria-targeted molecular imaging can meet these needs. The present study was aimed at the in vivo evaluation of two ^18^F-vancomycin-based PET tracers, for detection of deep-seated Gram-positive bacterial infections. These tracers were bench-marked against the current standard of care, [^18^F]FDG.

**Methods:**

The potential of [^18^F]BODIPY-FL-vancomycin and [^18^F]PQ-VE1-vancomycin ([4+2]photocycloadduct of 9,10-phenanthrenequinone-vancomycin and [^18^F]fluorinated vinyl ether) to distinguish bacterial infections from sterile inflammation was evaluated in a murine myositis model. Tracer specificity was assessed by infecting mice either with the Gram-positive bacterium *Staphylococcus aureus* (*n* = 12) or the Gram-negative bacterium *Escherichia coli* (*n* = 12). The contralateral leg was injected with Cytodex beads to induce sterile inflammation, or with phosphate-buffered saline for control. In parallel, mice were imaged with [^18^F]FDG (*n* = 12). Dynamic positron emission tomography (PET) measurements, biodistribution analyses, and immunohistopathology were performed to determine tracer distribution and bacterial burden.

**Results:**

Both ^18^F-vancomycin-PET tracers accumulated at sites of infection, but not at sites of sterile inflammation, in contrast to [^18^F]FDG. The tracers exhibited distinct biodistribution profiles, with [^18^F]BODIPY-FL-vancomycin being cleared more rapidly. Both ^18^F-vancomycin-PET tracers displayed significant target to non-target ratios of 2.95 for [^18^F]BODIPY-FL-vancomycin and 1.48 for [^18^F]PQ-VE1-vancomycin.

**Conclusion:**

Vancomycin-based PET is a potentially attractive approach to distinguish Gram-positive bacterial infections from sterile inflammation.

**Supplementary Information:**

The online version contains supplementary material available at 10.1007/s00259-024-06997-z.

## Introduction

Bacterial infections, especially those caused by antibiotic resistant bacteria, are a major cause of patient morbidity and mortality, and they represent a serious global health threat [1]. To effectively treat such infections and to improve patient outcome through tailored antibiotic therapy, an early and accurate diagnosis is of critical importance [2]. Unfortunately, this is highly challenging in case of deep-seated infections that are not readily accessible for the collection of samples/biopsies and subsequent diagnostic microbiological processing.

Today, evidence for deep-seated infections can be obtained through anatomical imaging modalities, such as computed tomography (CT) and magnetic resonance imaging (MRI), or through functional imaging modalities such as positron emission tomography (PET) and single-photon emission computed tomography (SPECT) [3, 4]. Fluorescence- and optoacoustic imaging, currently in preclinical assessment for diagnosing infection, may be included in future clinical procedures [5]. In particular, PET/CT with [^18^F]fluorodeoxyglucose ([^18^F]FDG) is a highly sensitive approach to non-invasively image deep-seated infections and the associated inflammatory responses of the human body [3, 6]. This relates to the fact that the [^18^F]FDG tracer will accumulate in cells with increased glucose metabolism, such as immune cells that are recruited to sites of infection [6–8]. Although bacterial pathogens can accumulate [^18^F]FDG, possibly contributing to the infection-specific component of the PET signal [9], the afore-mentioned anatomical and functional imaging modalities will only provide indirect evidence of infection through secondary symptoms, such as leukocyte infiltration, structural changes in organs and tissues, increased blood flow, or increased metabolic activity [10]. Moreover, [^18^F]FDG-PET cannot distinguish infection from wound healing or sterile inflammation caused by a foreign body response of the immune system [11]. Consequently, there is a pressing need for bacteria-targeted imaging approaches that directly visualize the causative agents of infection [11, 12].

Direct detection of invasive bacteria can be achieved through conjugation of a molecule that is specifically bound or internalized by a bacterial pathogen (e.g. an antibiotic, antibody or metabolizable compound) with a molecule that can readily be detected (e.g. a radionuclide or fluorophore) [13, 14]. This approach is exemplified by the development of radiolabelled antibiotic-based tracers for Gram-positive and Gram-negative bacteria [12, 15]. A highly promising candidate for bacterial infection imaging is the antibiotic vancomycin, a glycopeptide that selectively binds the d-Alanyl-d-Alanyl motif in bacterial peptidoglycan precursors [16]. The application potential of vancomycin as a targeting molecule for bacterial infection imaging has been demonstrated with the optical imaging tracer vancomycin-IRDye800CW in a murine myositis model [5], a murine spinal implant infection model [17] and a human post-mortem implant model [18]. In addition, vancomycin-IRDye800CW was also successfully applied for the ex vivo imaging of Gram-positive bacterial biofilms on extracted osteosynthesis devices from patients with fracture-related infections [19]. Subsequent efforts to label vancomycin with fluorophores by others confirmed these findings [20–22]. Notwithstanding these encouraging results, the drawbacks of optical imaging, such as limited light penetration through human tissue (± 1 cm) and tissue autofluorescence, underscore the need for other imaging modalities [23]. Better signal penetration through tissue of up to ± 8 cm can be achieved by conjugating vancomycin with fluorophores that allow photoacoustic imaging [24], but a radiolabelled vancomycin conjugate that can be applied for PET/CT would be preferable.

**Fig. 1:**
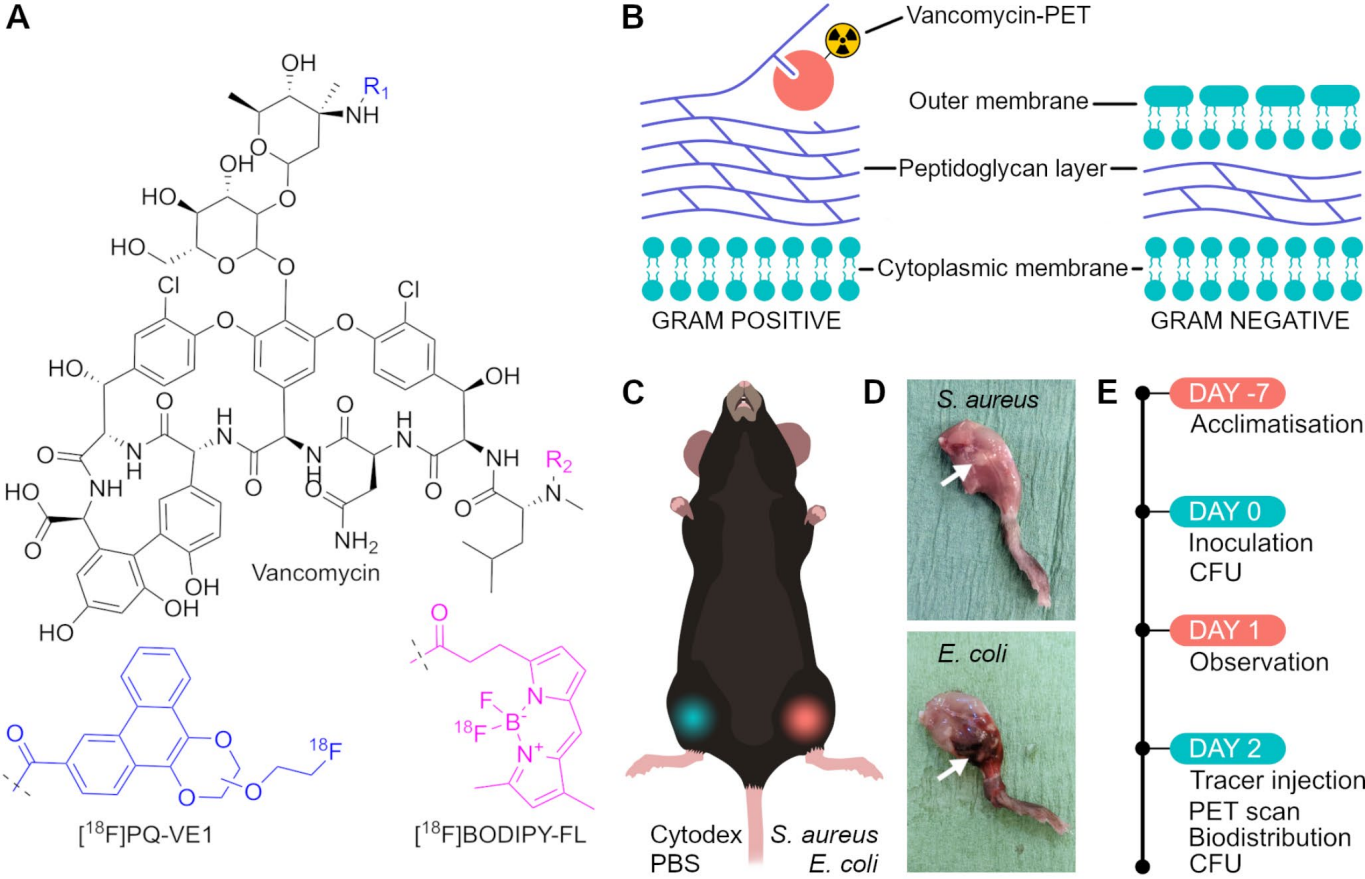
Experimental set-up and timeline of the murine myositis infection model. (**A**) Chemical structures of [^18^F]PQ-VE1-vancomycin and [^18^F]BODIPY-FL-vancomycin. [^18^F]PQ-VE1 is conjugated to the primary amine (R_1_) of vancomycin, whilst [^18^F]BODIPY-FL is conjugated to the secondary amine (R_2_) of vancomycin. (**B**) Simplified model of the cell envelopes of Gram-positive and Gram-negative bacteria. ^18^F-vancomycin-PET tracers will bind to d-Ala-d-Ala moieties in the peptidoglycan of Gram-positive bacteria, whereas binding is precluded by the outer membrane of Gram-negative bacteria. (**C**) In the murine left hind leg, infection was induced with *S. aureus* or *E. coli*. The right hind leg was injected with sterile Cytodex beads to induce inflammation or PBS for control. (**D**) Representative images of infected hind legs illustrating typical symptoms of infection by *S. aureus* or *E. coli*. Arrows indicate extensive haematoma and extravasation due to *E. coli* infection and indurated tissue due to *S. aureus* infection. (**E**) Timeline of the experimental procedures

Recently, we developed a set of ^18^F-labelled vancomycin conjugates [25, 26]. Two of these conjugates, [^18^F]BODIPY-FL-vancomycin and [^18^F]PQ-VE1-vancomycin (Fig. 1A), were stable in vitro, showed low non-target uptake in healthy animals and showed specificity towards Gram-positive bacteria in vitro. However, it was not yet known to what extent they allow detection of invasive bacteria in vivo. Therefore, the aims of the present study were to investigate the in vivo target specificity of [^18^F]BODIPY-FL-vancomycin and [^18^F]PQ-VE1-vancomycin in a murine myositis infection model, and to benchmark them against [^18^F]FDG as the current clinical standard for infection imaging.

## Materials and methods

### Bacterial strains and growth conditions

*Staphylococcus aureus* strain Xen36 [27] (PerkinElmer, Waltham, United States) and *Escherichia coli* strain Xen14 [28] were used for in vivo infection experiments. Bacteria from a -80 °C glycerol stock were streaked on tryptic soy agar (TSA) or lysogeny broth (LB) agar with 200 μg/mL kanamycin, and grown for 24 h at 37 °C. Subsequently, a single colony was selected, and 4 mL of tryptic soy broth (TSB) or LB with 200 μg/mL kanamycin were inoculated for overnight culture at 37 °C and 250 revolutions per min (rpm). On the day of the experiment, 10 mL of fresh medium was inoculated to an optical density at 600 nm (OD_600_) of 0.01, and culturing was continued for 2.5–3 h to an OD_600_ of ± 0.8 (37 °C, 250 rpm). Bacteria were then collected by centrifugation (1 min, 10.000 rpm), and the bacterial pellets were resuspended in phosphate-buffered saline (PBS) to produce bacterial inoculums for murine infection of approximately 5 × 10^7^ colony-forming units (CFU) per 25 μL. CFU counts of the inoculums pre- and post-inoculation was performed by serial dilution, plating on Mueller Hinton agar (MHA, Oxoid, Hampton, United States), incubation of the plates for 24 h at 37 °C and colony-counting. Similarly, post-termination tissue homogenates were serially diluted and plated on MHA. After 24 h incubation at 37 °C, bacterial colonies were counted and the CFUs per gram of tissue were calculated.

For in vitro control experiments to test tracer binding, a biofilm-adapted *E. coli* ATCC 25922 strain and the *S. aureus* NCTC 8325 strain were used.

### Tracers

[^18^F]BODIPY-FL-vancomycin [26] and [^18^F]PQ-VE1-vancomycin [25] were synthesized as previously described. [^18^F]FDG was produced in-house and used from stocks of 200 MBq in 5 mL 0.9% saline. Both vancomycin-based PET tracers were formulated using an Oasis HLB cartridge, using absolute EtOH for product elution. The final EtOH concentration was brought below 10% using 0.9% saline solution. Molar activity for [^18^F]BODIPY-FL-vancomycin and [^18^F]PQ-VE1-vancomyin was determined at 5.35 ± 3.91 GBq/μmol and 415 ± 210 GBq/μmol, respectively [26].

### Mice

Thirty-six 9-week-old male C57BL/6 wild-type mice (Charles River, Wilmington, USA) were utilized for infection experiments (Table S1). Animal housing and care were in accordance with the institutional protocol (AVD10500202114768). Specifically, four mice were housed per cage, and they were supplied with chow and water *ad libitum*. Animal rooms were kept on a 12-h light/dark cycle. No experiments were performed during the dark phase. Animals were observed once a week prior to the start of the experiments. During the experiments, animal behaviour, locomotion and weight were monitored daily (Table S1).

### General study design

A murine myositis model was established to compare the performance of the two vancomycin-based PET tracers in the in vivo detection of invasive Gram-positive bacteria. For control, [^18^F]FDG was used to image infection and inflammation. Mice were randomly divided over equally sized groups and myositis was induced in their left hindleg by injection of the Gram-positive bacterium *S. aureus* (*n* = 6) or the Gram-negative bacterium *E. coli* (*n* = 6). For control, the right hindleg was injected with sterile PBS (*n* = 6) or sterile Cytodex beads (Cytiva, Marlborough, USA) (*n* = 6). Thus, 12 animals were used per investigated tracer, totalling 36 animals. Each group was subsequently injected with a PET tracer and underwent PET scanning. In addition, a biodistribution analysis was performed to determine tracer accumulation in relevant organs. Animals were excluded from the study if weight loss was greater than 15%, or if the animal died. Investigators were not blinded to the (infection) status of the animal.

### In vivo experimental procedures

During bacterial inoculation and PET imaging, the mice were anesthetized with a gas mixture of isoflurane and oxygen (5% for induction, 1.0-2.5% for maintenance). Meanwhile, body temperature was regulated using a heating pad. Bacterial inoculation was performed by injection of 25 μL with 5 × 10^7^ CFU of either *S. aureus* or *E. coli* in the left hamstring, using a 0.3 mL syringe with a 32G needle. To induce sterile inflammation in the contralateral hamstring, 25 μL of either sterile PBS or Cytodex beads (1.0 mg in 25 μL, Cytodex 1 microcarrier beads, 60–87 μm, Sigma Aldrich, Saint Louis, USA) were injected using a 1 mL syringe with a 27G needle. As previously described, the injected Cytodex beads elicit a foreign body response that results in sterile inflammation [5]. Furthermore, one dose of buprenorphine (0.05 mg/kg) was given subcutaneously as analgesic. Bacterial inoculation was assessed by CFU-counting of the inoculum as above. The animals were observed for 24 h post-inoculation, and weight loss was monitored. After 48 h post-inoculation, PET imaging was performed. All PET-tracers, in 100–200 μL saline, were injected retro-orbitally. Specifically, this involved 2.48 MBq ± 0.34 (0.80 ng ± 0.10) for BODIPY-FL-vancomycin, 1.42 MBq ± 1.01 (0.006 ng ± 0.004) for [^18^F]PQ-VE1-vancomycin, and 1.40 MBq ± 0.61 for [^18^F]FDG. This administration route was chosen to avoid possible spill-over effects, because it creates the largest possible distance between the site of tracer injection and the induced myositis. Immediately after tracer administration, mice were positioned in a small animal microPET scanner (Focus220, Siemens Medical Solutions, Malvern, USA) and a 50-min dynamic PET scan was started, followed by a 10-min transmission scan with a ^57^Co point source.

### Ex vivo analysis

After completion of PET-scanning, i.e. 60 min after tracer administration, the mice were sacrificed by cervical dislocation, and tissue and organ samples were collected for biodistribution analysis. This was done, because the in vivo PET data provide information on the local tracer intensity (individual voxel values) and the area of tracer accumulation (ROI volume) at sites of infection or inflammation, but not on the total tracer activity accumulated per organ or tissue. Radioactivity of the collected samples in counts per min (cpm) was measured using a gamma counter (Wizard2, PerkinElmer, USA). The target to non-target ratios (T/NT) were calculated by dividing the activity (%ID/g) in the hind leg by the background activity in healthy muscle tissue from the front limb. For assessment of tracer accumulation in the hind legs, a multi-step approach was followed to allow for histological and CFU analysis of tissue without impacting activity measurements. First, the hind leg was fully excised, and radioactivity was measured. Subsequently, a 3 mm punch biopsy was taken from the hamstring and kept in 4% paraformaldehyde (PFA) for 24 h at room temperature. Thereafter the sample was washed in PBS and stored at 4 °C until further processing for histology. The remaining hamstring was excised and placed in a screwcap Eppendorf tube with MHB and 100 mg of 1 mm glass beads (Biospec, Bartlesville, USA) plus a single 3 mm glass bead for tissue homogenization. The separate tissue samples from the hind leg (i.e. biopsy, excised hamstring, and remaining leg tissue) were then measured with the gamma counter for a second time. The excised hamstrings in MHB were homogenized using a tissue homogenizer (Precellys 24, Bertin, Montigny-le-Bretonneux, France) in 3 cycles of 30 s at 5000 rpm. Thereafter, the homogenized samples were centrifuged for 1 min at 5000 rpm to separate cell debris and supernatant. A fraction of 20 μL supernatant was taken for CFU-counting.

### PET data analysis

List-mode data from PET emission scans were reconstructed into 20 frames (6 × 10, 4 × 30, 2 × 60, 1 × 120, 1 × 180, 4 × 300, 2 × 600 s). After normalization, attenuation correction and decay correction, emission sinograms were iteratively reconstructed (OSEM2D-Z1-SC-256). PET images were analysed using Amide (version 1.0.6). Regions of interest (ROIs) were drawn around the hind legs, and radioactivity concentrations were calculated from these ROIs. Tissue radioactivity was corrected for injected dose, decay and body weight and expressed as standardized uptake value (SUV).

### Histology of infected muscle tissue

For histology, several tissue slides from each PFA-fixed punch biopsy were stained with haematoxylin and eosin (H&E). The remaining unstained tissue slides were deparaffinized by dissolving the paraffin in warm xylene (35 °C), followed by xylene at room temperature, isopropanol, 100% EtOH, 96% EtOH, 70% EtOH, and a final wash in Milli-Q (MQ) water. All washing steps were performed at room temperature for 5 min. The deparaffinized samples were stained with vancomycin-BODIPY-FL (Invitrogen, Waltham, United States), and 4′,6-diamidino-2-phenylindole (DAPI). For staining with vancomycin-BODIPY-FL, tissue samples were incubated with 2 μL probe (67 μM) for 30 min at 37 °C and washed twice with sterile MQ water. Thereafter, DAPI staining was repeated. Images were collected using a Leica DM5500B microscope (Leica, Wetzlar, Germany), equipped with an X-cite 200DC laser (Excelitas, Mississauga, Canada), and a Leica DFC365FX CCD microscope camera module. H&E-stained tissue samples were analysed at 100x magnification (Leica PL Fluotar 10x) and images were captured using a smartphone device. Fluorescence images of tissue samples stained with vancomycin-BODIPY-FL and DAPI were collected at 400x magnification (Leica PL FLUOTAR 40x) using the Leica DFC365FX CCD microscope camera module. Image analysis was performed using ImageJ (version 1.54d).

### Statistical analyses

All data are presented as mean ± standard deviation (SD), unless otherwise stated. Data analysis was performed with R version 4.3.1 (R core team 2022, Vienna, Austria) using R studio version 2023.06.0. Statistical analyses were performed using the Mann Whitney test and the Kruskal-Wallis test for non-parametric data. *P*-values < 0.05 were considered significant.

## Results

To evaluate whether [^18^F]BODIPY-FL-vancomycin and [^18^F]PQ-VE1-vancomycin can accurately localize Gram-positive bacteria at the site of infection, and differentiate infection from sterile inflammation, a murine myositis model was implemented (Fig. 1). Inoculation of the mice with a bacterial load of 5 × 10^7^ CFU/mL led to persisting local infections, as verified by culturing of the hind leg muscle tissue homogenates post-termination (Figure S1). Furthermore, indicators of systemic infection, such as weight loss of more than 10%, and behavioral or physical changes, such as lethargy, shivering or piloerection, were absent during the observational period. This was in line with the finding that no colonization of non-infected tissue was detected through culturing of contralateral hind leg muscle tissue. Notably, the visual presentation of infections differed for *S. aureus* and *E. coli*, even though the bacterial loads were comparable (Fig. 1D). In legs infected with *E. coli*, extensive haematoma formation was observed. Yet, the infected legs allowed a relatively well conserved range of motion. In contrast, legs infected with *S. aureus* showed minimal haematoma formation but, instead, discolored and indurated tissue was observed at the injection site, leading to severe hind leg rigidity.

Two days after inoculation, either [^18^F]BODIPY-FL-vancomycin, [^18^F]PQ-VE1-vancomycin (Fig. 1A), or [^18^F]FDG was retro-orbitally administered to the mice (Fig. 1E). Immediately after tracer administration, a 50-min dynamic PET-scan was performed, followed by a 10-min transmission scan (Fig. 1E).

**Fig. 2:**
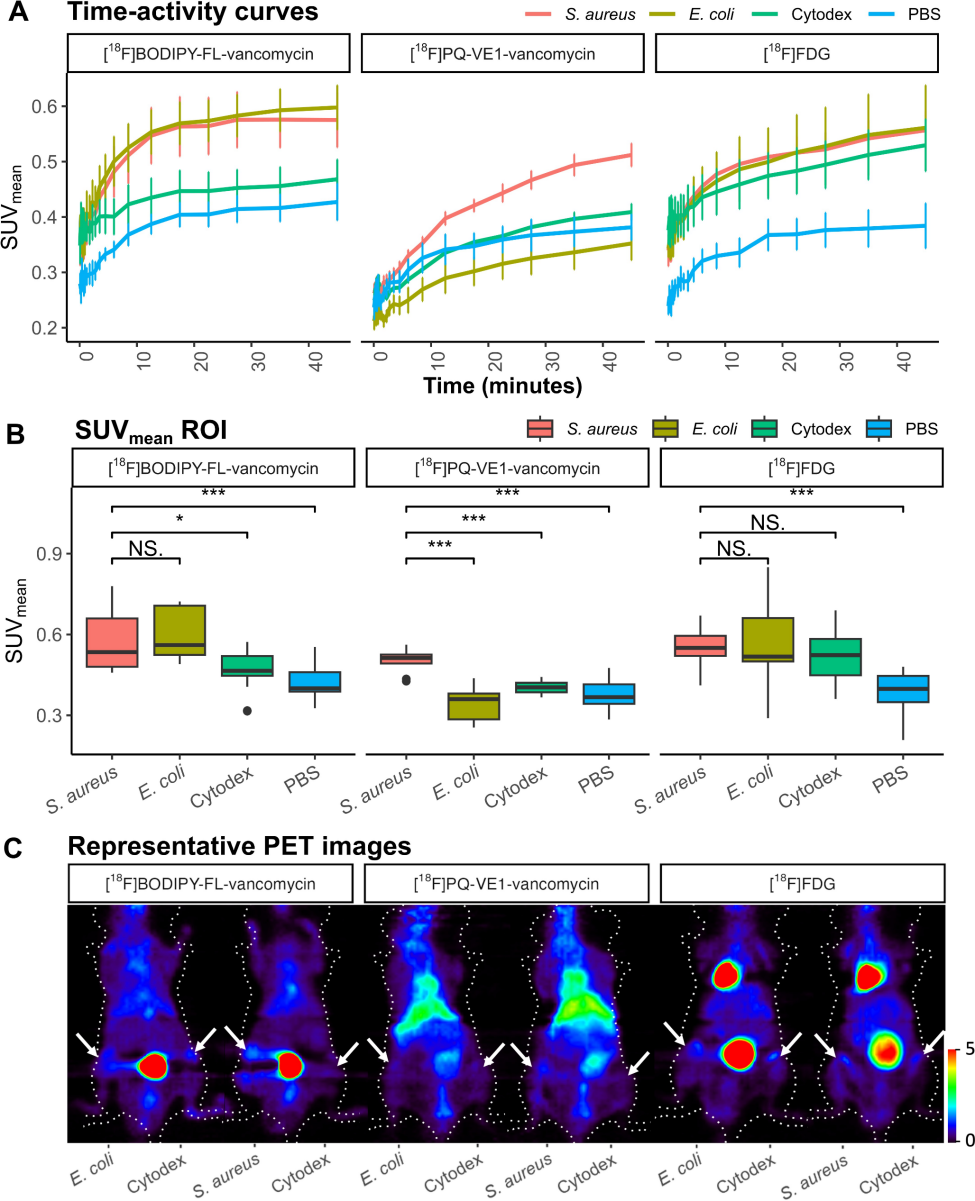
PET imaging of bacterial infection with [^18^F]BODIPY-FL-vancomycin, [^18^F]PQ-VE1-vancomycin or [^18^F]FDG. Mice with *S. aureus* or *E. coli*-induced myositis, or Cytodex bead-induced inflammation were monitored for tracer accumulation by 50-min dynamic emission scans and 10-min transmission scans in a microPET imaging system, directly post-administration of the respective tracer. PBS was injected for control. (**A**) Time activity curves for all PET tracers in the different ROIs; red, *S. aureus* infection; brown, *E. coli* infection; green Cytodex bead induced inflammation; blue, PBS control. Activity was measured as the SUV_mean_ and expressed as the mean ± SD. The list-mode data from the emission scan were reconstructed into 20 frames. (**B**) Tracer uptake in hind legs presented as ROIs for both ^18^F-vancomycin-PET tracers and [^18^F]FDG upon analysis of the last two 10-min frames (30 min post-injection) from the dynamic emission scan. Data is represented as the median with the interquartile range (IQR). Wilcoxon signed rank test: *, *p* < 0.05; **, *p* < 0.01, ***, *p* < 0.001. (**C**) Representative PET images (coronal view) of two animals per tracer, showing tracer signals in areas of *E. coli* or *S. aureus* infection in the left hind leg versus Cytodex bead-induced inflammation contralaterally. Arrows indicate the sites of injection of bacteria, beads or PBS. The colour legend on the right indicates the SUV_mean_

Regions of interest (ROIs) were delineated from PET scan data and mean standardized uptake values (SUV_mean_) were calculated (Fig. 2). During dynamic emission PET scanning, [^18^F]BODIPY-FL-vancomycin uptake reached a plateau around 20 min post tracer administration for all conditions (Fig. 2A). In contrast, the PET signals in the ROIs for [^18^F]PQ-VE1-vancomycin continued to increase over time. When further comparing infection conditions, it was observed that sites of *S. aureus* infection showed high SUV_mean_ values for both ^18^F-vancomycin-PET tracers (Fig. 2B). Moreover, a clear distinction could be made between infected legs, legs presenting Cytodex bead-induced inflammation (Cytodex) or PBS controls. In contrast, [^18^F]FDG did not discriminate between infections caused by *S. aureus* or *E. coli* and Cytodex bead-induced inflammation. Interestingly, administration of [^18^F]BODIPY-FL-vancomycin also resulted in high SUV_mean_ values for *E. coli* infection. This is in contrast to the administration of [^18^F]PQ-VE1-vancomycin, which resulted in selective accumulation in *S. aureus* infection. This high uptake of [^18^F]BODIPY-FL-vancomycin in *E. coli*-infected tissue cannot be attributed to differences in ROI sizes for the *S. aureus* and *E. coli* infection groups, as the ROI sizes were comparable (Figure S2). Furthermore, high bladder signals were observed in mice injected with [^18^F]BODIPY-FL-vancomycin and [^18^F]FDG, whereas substantial signal in the liver region was observed in mice injected with [^18^F]PQ-VE1-vancomycin, with less tracer accumulating in the bladder (Fig. 2C).

**Fig. 3:**
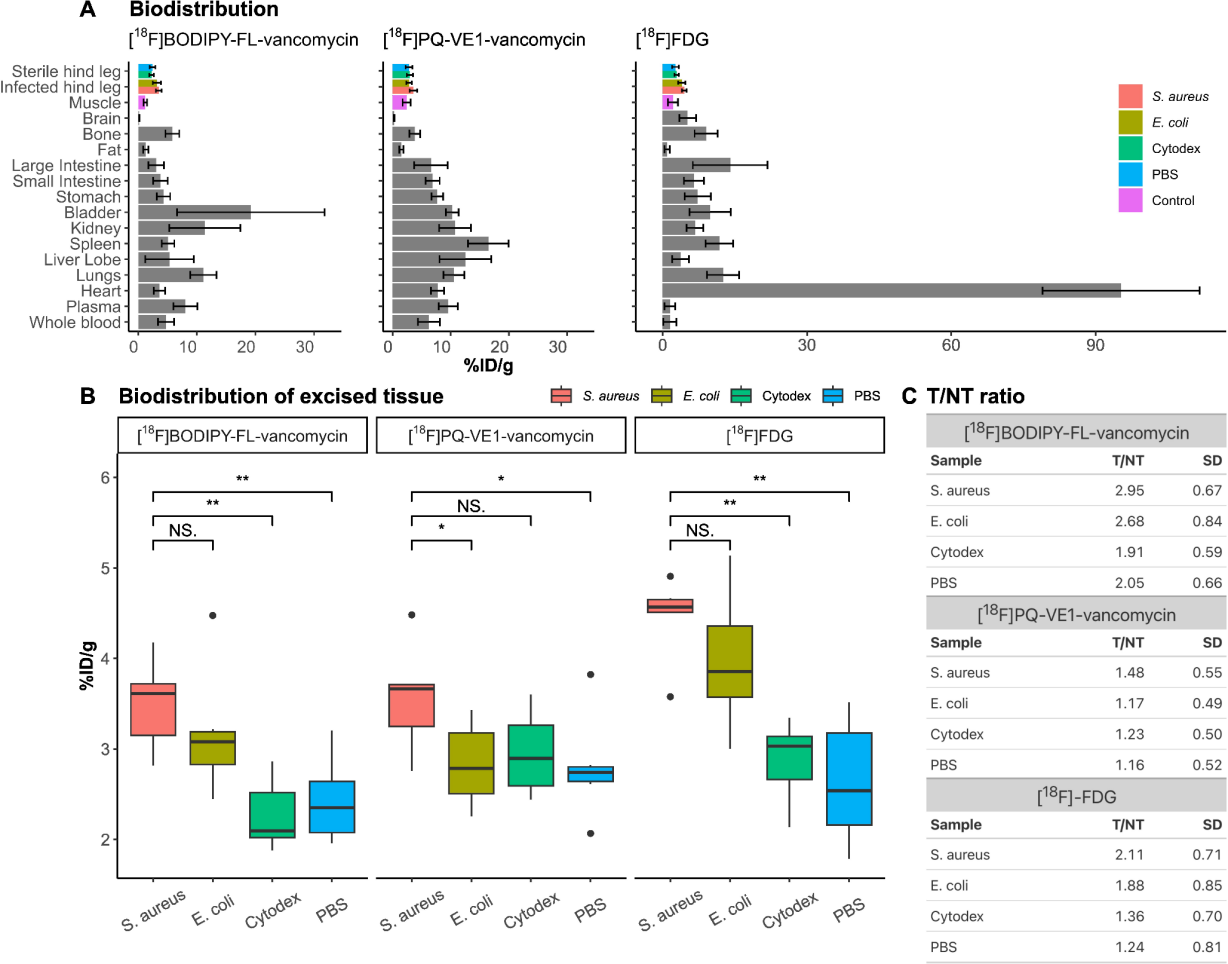
Biodistribution of [^18^F]BODIPY-FL-vancomycin and [^18^F]PQ-VE1-vancomycin. (**A**) Tracer distribution at 60 min post-injection for both ^18^F-vancomycin-based PET tracers. Radioactivity was measured as percentage of the injected dose per gram tissue (%ID/g), expressed as mean ± SD. Highlighted in colour are the hind leg tracer uptake, as well as control muscle tissue derived from the front limb (purple). “Sterile hind leg” is a split-bar representing the PBS control (blue) and Cytodex bead-induced inflammation (green). ‘Infected hind leg” represents *S. aureus* (red) or *E. coli* infection (brown). (**B**) Magnified representation of uptake of both ^18^F-vancomycin-PET tracers and [^18^F]FDG in hind legs with *S. aureus*- or *E. coli*-induced myositis, Cytodex bead-induced inflammation or control legs injected with PBS. The data is presented as the median with IQR. Wilcoxon signed rank test: *, *p* < 0.05; **, *p* < 0.01. (**C**) Target to non-target (T/NT) ratios expressed as mean ± SD for the three PET tracers. Non-target signal was defined as healthy muscle tissue as in (A)

Directly after PET scanning, the biodistribution of [^18^F]BODIPY-FL-vancomycin, [^18^F]PQ-VE1-vancomycin and [^18^F]FDG was assessed. The data presented in Fig. 3A indicate a high accumulation in the kidneys, spleen, and lungs for both vancomycin-PET tracers. This was most pronounced for the [^18^F]PQ-VE1-vancomycin tracer, where the overall signals in these organs were higher than observed for the [^18^F]BODIPY-FL-vancomycin tracer, indicating a slower clearance rate of [^18^F]PQ-VE1-vancomycin (Table S2). Furthermore, in line with the known pharmacokinetics of vancomycin, strong signals of [^18^F]BODIPY-FL-vancomycin in the bladder and urine were indicative of renal clearance, whereas increased signal of [^18^F]PQ-VE1-vancomycin in the liver and intestines suggested predominantly hepatobiliary clearance.

The highest signals of all three PET tracers were observed at sites of *S. aureus* infection (Fig. 3B). Specifically, the T/NT ratios at sites of *S. aureus* infection were highest in the case of [^18^F]BODIPY-FL-vancomycin (Fig. 3C). Furthermore, the non-target signals observed for [^18^F]PQ-VE1-vancomycin were relatively elevated compared to [^18^F]BODIPY-FL-vancomycin, in line with the idea that clearance was slower for [^18^F]PQ-VE1-vancomycin than for [^18^F]BODIPY-FL-vancomycin. Lastly, increased uptake of [^18^F]FDG was observed both for sites of *S. aureus* and *E. coli* infection, and to a lesser degree also at sites of Cytodex bead-induced inflammation.

Intriguingly, we also observed accumulation of [^18^F]BODIPY-FL-vancomycin at sites of *E. coli* infection, which was not detectable for [^18^F]PQ-VE1-vancomycin (Fig. 3B). This phenomenon was not apparent in our previous in vitro experiments where [^18^F]BODIPY-FL-vancomycin was shown to bind to planktonic *S. aureus* bacteria, but not to planktonic *E. coli* bacteria [26]. This species-specific difference in binding of vancomycin-based tracers relates to the fact that the peptidoglycan in the cell envelope of Gram-negative bacteria, like *E. coli*, is shielded by a vancomycin-impermeable outer membrane. Such an outer membrane is absent from Gram-positive bacteria, like *S. aureus*, which explains the preferential binding of vancomycin to these bacteria (Fig. 1B). However, it was conceivable that, during infection, the outer membrane of at least some *E. coli* bacteria was more permeable for [^18^F]BODIPY-FL-vancomycin, or that this tracer was bound by dead bacteria, or peptidoglycan fragments in bacterial debris [29, 30]. To investigate these possibilities, in vitro tracer binding experiments were performed with *E. coli* and *S. aureus* bacteria derived from planktonic cultures or sonicated biofilms and with heat-killed bacteria (Figure S3; Supplemental Methods). In this in vitro set-up, both ^18^F-vancomycin-PET tracers demonstrated high and specific binding to *S. aureus*, while none of the *E. coli* preparations showed notable tracer binding. This implies that the in vivo conditions of murine myositis specifically favour binding of [^18^F]BODIPY-FL-vancomycin to *E. coli* by an undefined mechanism.

**Fig. 4:**
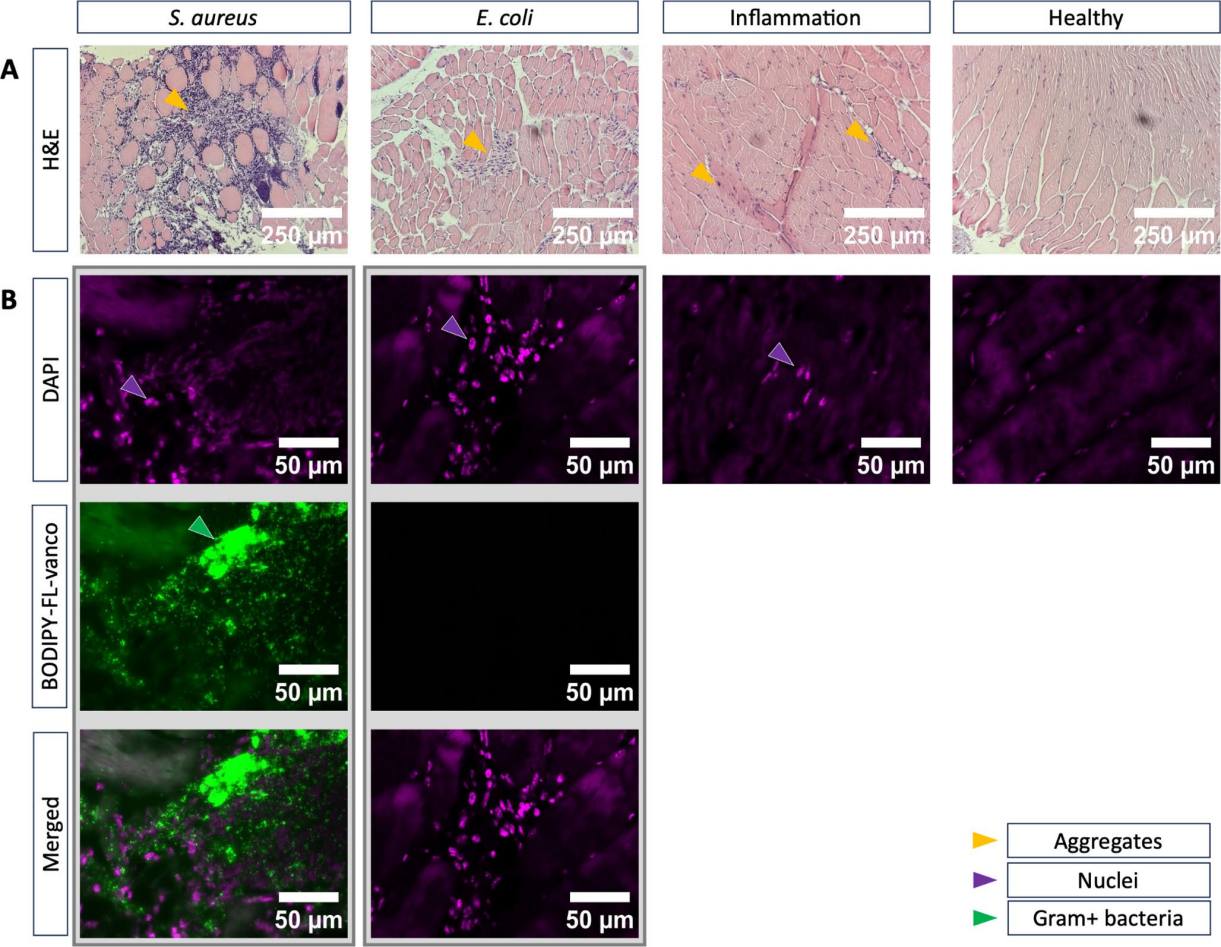
Histological analysis of tissue biopsies from mice with bacterial or sterile myositis. (**A**) H&E-stained tissue biopsies collected from hind legs of mice with myositis due to infection with *S. aureus* or *E. coli*, or due to injection of Cytodex beads. Large aggregates of bacteria and immune cells can be observed in *S. aureus*-infected tissue. Immune cells are marked with yellow arrowheads. Please note that injected Cytodex beads were not visualized in the Inflammation condition, which is due to the relatively low numbers of injected beads compared to bacteria and the fact that, unlike the injected bacteria, the beads will not replicate upon injection. The scale bar indicates 250 μm. (**B**) Immune cell infiltration in infected or inflamed tissue was visualized by DAPI-staining of the respective nuclei (purple arrowheads). *S. aureus* bacteria were stained with BODIPY-FL-vancomycin (green arrowheads). BODIPY-FL-vancomycin signals were completely absent from *E. coli*-infected tissues. Scale bars indicate 50 μm

To verify the presence of bacteria in the hind legs of infected mice at the time of tracer administration and PET analysis, 3 mm punch biopsies were collected, fixated and used for histology. In the BODIPY-FL-vancomycin-stained tissue samples of *S. aureus-*infected animals, Gram-positive cocci were detected, confirming the staphylococcal infection (Fig. 4). Furthermore, a strong immune response was observed, as highlighted by the DAPI-stained nuclei of intramuscular immune cells. A similar immune response was detected in tissue samples from *E. coli-*infected mice. As expected, no BODIPY-FL-vancomycin signal was observed in the *E. coli*-infected tissue. It was not possible to pinpoint Cytodex beads in the murine tissues, which presumably relates to the relatively low numbers of injected beads. Nonetheless increased levels of intramuscular immune cells were detected, compared to healthy controls, verifying that injection of Cytodex beads had caused inflammation.

## Discussion

To allow for rapid and non-invasive diagnosis of bacterial infections, and thereby guide clinical decision-making, PET tracers that specifically target bacteria are urgently needed [31]. In our present preclinical study, we show the successful bacteria-targeted imaging of staphylococcal infection with two vancomycin-based PET tracers in a murine myositis model. In particular, vancomycin-based PET was successfully applied to distinguish bacterial infection from sterile inflammation. In contrast, the commonly applied [^18^F]FDG tracer showed similar levels of uptake in both conditions, underscoring the notion that it does not distinguish infection from sterile inflammation. The latter is expected, as elevated glucose metabolism is, by itself, not a discriminating feature of infection. Our [^18^F]BODIPY-FL-vancomycin tracer showed a 3-fold higher *S. aureus* infection-specific signal compared to background. Moreover, the [^18^F]PQ-VE1-vancomycin tracer demonstrated consistent specificity towards staphylococcal infection upon analysis of the in vivo PET data, its biodistribution and its in vitro binding to bacteria.

Interestingly, [^18^F]BODIPY-FL-vancomycin was found to accumulate both at sites of *S. aureus* and *E. coli* infection. This is in contrast with this tracer’s in vitro binding specificity for Gram-positive bacteria including *S. aureus*, and the known specificity of the antibiotic vancomycin for Gram-positive but not Gram-negative bacteria [32]. Recently, however, Zhang & co-workers described the synthesis and in vitro binding of a vancomycin-derivative to *E. coli* mutants that exhibit a leaky cell wall [21]. The authors hypothesized that this phenomenon might be due to the conjugation of a small fluorophore with only minimal electronic charge to vancomycin. It is conceivable that a similar behaviour was observed for [^18^F]BODIPY-FL-vancomycin in *E. coli*-induced myositis, as the molecular weight of this tracer is merely increased with 290 Da compared to vancomycin. However, the specificity of [^18^F]BODIPY-FL-vancomycin for *E. coli* was only observed in vivo, suggesting a role for an as of yet unidentified murine factor in permeabilizing the outer membrane and giving access to the *E. coli* peptidoglycan, or allowing binding of [^18^F]BODIPY-FL-vancomycin to the bacterial cell surface. Irrespective of this, [^18^F]PQ-VE1-vancomycin features similar characteristics as [^18^F]BODIPY-FL-vancomycin without showing an enhanced in vivo signal in the context of an *E. coli* infection. Though rather unlikely, another possible explanation for the high *E. coli*-targeted in vivo signal observed with [^18^F]BODIPY-FL-vancomycin could be that all six animals from this experimental group mounted a higher immune response than the *E. coli*-infected animals in the experimental groups where infection was imaged with [^18^F]PQ-VE1-vancomycin. If so, the higher inflammation might result in greater tracer extravasation and slower clearance of the non-specific signal. Unfortunately, it is difficult to retrospectively quantify the levels of induced inflammation, since our histological analyses yield only qualitative information about the inflammatory status of the inspected tissues. Importantly, the finding that [^18^F]BODIPY-FL-vancomycin is capable of detecting an *E. coli* infection in vivo may as well be perceived as an advantage. However, it also underlines the need for in vivo infection models with a closer resemblance to the clinical situation where the behaviour of tracers may be different from their behaviour in vitro.

From the present time-activity curves, it can be deduced that a longer period between tracer injection and PET scanning is likely to improve the T/NT ratios, at least for [^18^F]PQ-VE1-vancomycin. The relatively low T/NT ratios observed for [^18^F]PQ-VE1-vancomycin can be attributed to a higher background signal and a slower clearance rate compared to [^18^F]BODIPY-FL-vancomycin, rather than a reduced target signal. Altogether, the data indicate that for infection imaging with [^18^F]BODIPY-FL-vancomycin, the PET imaging timepoint at 60 min post tracer administration was appropriate, whereas for imaging with [^18^F]PQ-VE1-vancomycin a later time point might have been more optimal. Irrespective of the used tracer, enhancing the T/NT ratios will be important, because non-targeted tracer accumulation in the bone and any other background signals will complicate the targeted imaging of Gram-positive bacterial infections, especially in the case of bone infections like osteomyelitis. In this respect one has to take into account the relatively short half-life of ^18^F of (109.8 min), which necessitates imaging not too long after tracer injection. The latest EANM guideline recommends an [^18^F]FDG uptake time of 60 min in the case of inflammation/infection [33]. Lastly, we do not presently know the exact reason for the observed differences in the tissue clearance of our two vancomycin-based PET tracers but, possibly, they can be explained by differences in hydrophilicity and charge due to their respective conjugated prosthetic groups. Vancomycin is by itself a highly hydrophilic compound, but conjugation with BODIPY-FL or PQ-VE1 lowers its hydrophilicity (expressed as LogD_7.4_) from a theoretical value of −5.1 [34] to −1.78 and −0.76, respectively, as was previously determined experimentally [26]. Such a possible correlation between tissue clearance rates of tracers and their LogD_7.4_ was also previously described [35]. However, it remains remarkable that the conjugation with comparatively small prosthetic groups has such a distinct effect on the behaviour of our vancomycin-based tracers.

A potential pitfall of using antibiotics as imaging tracers is the routine administration of antibiotics in the clinical setting. Often, patients have been treated with multiple (broad spectrum) antibiotics before diagnostic imaging is performed [6]. However, our previous research showed that binding to Gram-positive bacteria was detectable up to, and over, MIC values in competition assays using both vancomycin-PET tracers and unlabelled vancomycin. This holds promise for bacterial infection imaging, even in patients pre-treated with vancomycin [26]. Furthermore, vancomycin resistance, for instance in *Enterococci*, is a clinical problem that could conceivably limit the applicability of vancomycin-based PET tracers [16]. However, we have previously shown that the optical tracer vancomycin-BODIPY FL binds both to clinical vancomycin resistant *vanA* and *vanB* isolates of *E. faecium*, showing that vancomycin resistance does not necessarily preclude vancomycin-based infection imaging [19].

Various efforts have been undertaken in the past to develop antibiotic-based radiolabelled tracers, starting with [^99m^Tc]ciprofloxacin (*Infecton*^®^). In the 1990’s, Langer et al. studied the PET tracer [^18^F]ciprofloxacin in patients with known infections, concluding that it was not suitable for bacteria-targeted imaging as its clearance from infected and uninfected tissues was comparable. This tempered the enthusiasm for antibiotic-based PET imaging for some time, but in recent years there has been a renewed interest in this topic and the development of antibiotic-based PET tracers for clinical translation [13, 15, 36, 37]. In particular, trimethoprim (TMP)-based PET tracers appear to be promising for bacterial infection imaging [38–40]. For both [^11^C]TMP and its analogue [^18^F]FP-TMP, binding to a broad range of Gram-positive and Gram-negative bacteria was reported in murine infection models as well as in patients [39, 40]. It will thus be interesting for future studies to investigate how the efficacy of our two vancomycin-based PET tracers in the detection of bacterial infections compares to that of [^18^F]FP-TMP.

In addition to antibiotic-based PET tracers, various other radiolabelled bacteria-targeting molecules have been developed [14, 41, 42]. For instance, infections by *Enterobacterales* may be targeted with 2-[^18^F]fluorodeoxysorbitol ([^18^F]FDS), which is specifically metabolized by this group of bacteria [43–46]. Similarly, [^11^C]*para*-aminobenzoic acid ([^11^C]PABA) and its analogue, [^18^F]PABA, which target the bacteria-specific folate synthesis pathway, may be used for infection imaging [47, 48]. However, these metabolic tracers are, by definition, dependent on metabolic activity for bacterial uptake, which imposes a challenge for the detection of bacteria with low metabolic activity as, for instance, encountered in biofilm-associated infections.

In our current study, two different ^18^F-vancomycin-PET tracers and [^18^F]FDG were benchmarked using the same protocols, thus allowing for a direct comparison of the performance of these three tracers in the detection of invasive bacteria and the distinction between infection and sterile inflammation. The results showed a superior performance of both vancomycin-based tracers over [^18^F]FDG in the distinction of bacterial infection and sterile inflammation. Yet, a limitation of our study is the lack of anatomical co-registration by MRI or CT. Hence, the ROIs in the PET images had to be drawn based on the radioactivity that accumulated at the approximated location of infection or inflammation in the murine hind legs. Accordingly, we decided to also measure total radioactivity in the entire hind leg with a gamma counter, which made a direct comparison with other data on the tracer biodistribution somewhat challenging. For future assessments, co-injection of a fluorescent tracer, or tissue stain, might help to circumvent this limitation, as it would allow better localization of the infection foci [49]. Inherent to a murine infection model, care should be taken when extrapolating the results to the clinical situation. The evaluation of vancomycin-based PET tracers in a myositis infection model is an exploratory first in vivo step in the development process. Ultimately, our aim is to provide a rapid and accurate diagnosis of Gram-positive bacterial infections, such as deep-seated soft tissue infections or biofilm-associated infections.

## Conclusion

Our present study provides the proof-of-principle that ^18^F-vancomycin-PET tracers can be added to the current toolbox of vancomycin-based tracers for the imaging of Gram-positive bacterial infections, as well as the distinction between bacterial infection and inflammation. These PET tracers offer the advantage of detecting deep-seated infections, which is not readily feasible with fluorescently labelled vancomycin-based tracers. Next steps towards testing the feasibility for clinical implementation of our vancomycin-PET tracers will involve their implementation in murine implant infection models and human post-mortem implant infection models. Although the current findings are encouraging, further investigation will be necessary to assess the clinical applicability of vancomycin-based PET tracers for bacteria-targeted molecular infection imaging.

## Electronic supplementary material

Below is the link to the electronic supplementary material.

Supplementary Material 1

## Data Availability

All data are provided with the manuscript.
