# Supplementary material for: Bacteria-targeted imaging using vancomycin-based positron emission tomography tracers can distinguish infection from sterile inflammation
Source: Eur J Nucl Med Mol Imaging. 2024 Nov 29;52(5):1878–89. doi: 10.1007/s00259-024-06997-z (PMC11928434; doi:10.1007/s00259-024-06997-z)
Supplement: Supplementary file 1 — Supplementary Material 1 [file 259_2024_6997_MOESM1_ESM.pdf]

Supplemental Materials

Bacteria-targeted imaging using vancomycin-based positron emission tomography tracers can distinguish infection from sterile inflammation

G. B. Spoelstra[1,*], L. M. Braams[2,*], F. F. A. IJpma[3], M. van Oosten[2], B. L. Feringa[4], W. Szymanski[5,6], P. H. Elsinga[1,†] and J. M. van Dijl[2,†,#]

[1] University of Groningen, University Medical Center Groningen, Department of Nuclear Medicine and Molecular Imaging, Hanzeplein 1, 9713GZ Groningen, The Netherlands;

[2] University of Groningen, University Medical Center Groningen, Department of Medical Microbiology and Infection Prevention, Hanzeplein 1, 9713GZ Groningen, The Netherlands;

[3] University of Groningen, University Medical Center Groningen, Department of Trauma Surgery, Hanzeplein 1, 9713GZ Groningen, The Netherlands;

[4] Stratingh Institute for Chemistry, University of Groningen, Nijenborgh 4, 9747 AG Groningen, The Netherlands

[5] University of Groningen, University Medical Center Groningen, Department of Radiology, Hanzeplein 1, 9713GZ Groningen, The Netherlands;

[6] University of Groningen, Groningen Research Institute of Pharmacy, Department of Medicinal Chemistry, Photopharmacology and Imaging, Antonius Deusinglaan 1, 9713 AV Groningen, The Netherlands

[#] **Corresponding author**: Prof. dr. Jan Maarten van Dijl, ORCID ID 0000-0002-5688-8438; Hanzeplein 1, 9700 RB Groningen, The Netherlands; email: j.m.van.dijl01@umcg.nl

[*,†] equal contributions

Content:

- Table S1: Animal characteristics
- Fig S1: CFU analysis of bacterial inoculums and harvested tissue
- Table S2: Tracer biodistribution
- Fig S2: Sizes of regions of interest (ROIs)
- Methods: *In vitro* experiments
- Fig S3: *In vitro* binding of ^{18}F-vancomycin-PET tracers.

Table S1: Animal characteristics

[a] Two animals in the group treated with [^{18}F]PQ-VE1-vancomycin were excluded due to mortality, which was not related to the induced infection.

SD = standard deviation

	[^{18}F]BODIPY-FL-vancomycin	[^{18}F]PQ-VE1-vancomycin	[^{18}F]FDG
	n = 12	n = 10a	n = 12
	Mean ± SD	Mean ± SD	Mean ± SD
Age (days) Scan day	60.6 ± 5.2	64.4 ± 5.3	61.6 ± 4.0
Weight (g) Inoculation day	23.9 ± 1.5	24.4 ± 1.5	24.0 ± 1.0
Weight (g) Scan day	21.6 ± 1.6	22.4 ± 1.5	22.0 ± 1.1

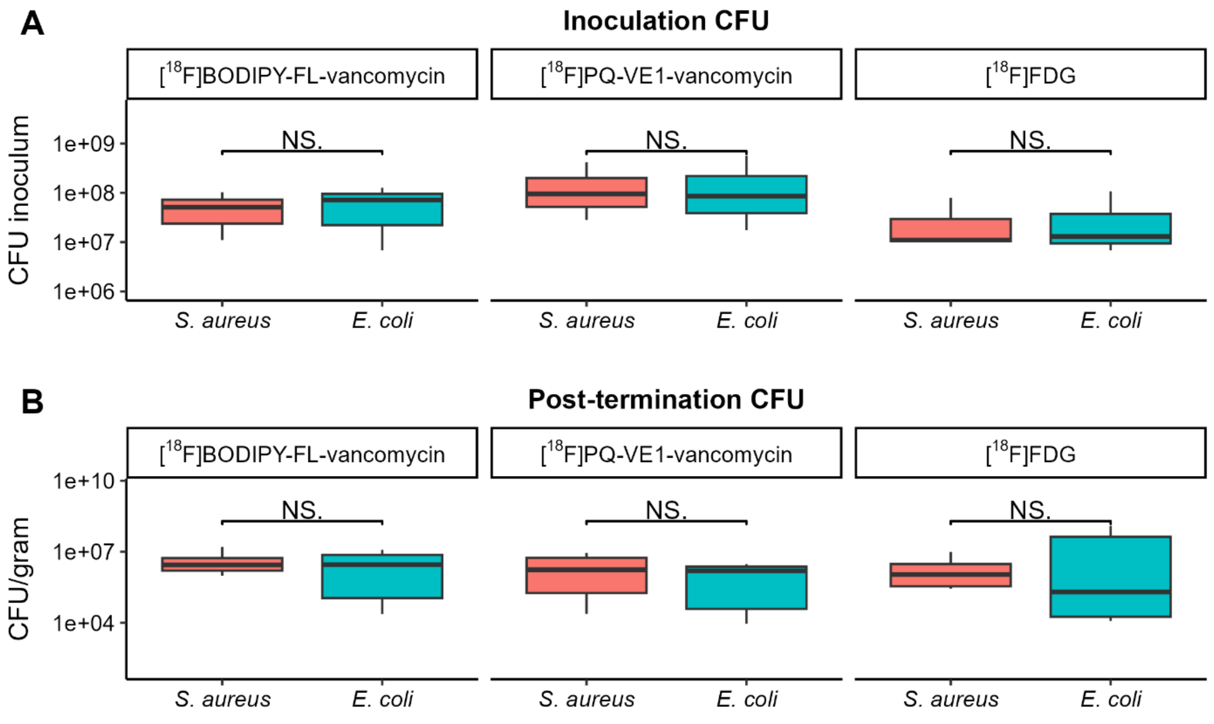

Figure S1: CFU analysis of bacterial inoculums and harvested tissue. Data is represented as median with IQR. Wilcoxon signed rank test; * = p < 0.05, ** = p < 0.01. **(A)** From the bacterial inoculum, a dilution series was made for CFU determination. No statistically significant differences were observed between the different inoculums. **(B)** Infected tissue was collected after termination of the animals and homogenized using a tissue homogenizer. From the tissue homogenate CFUs were determined and expressed as CFU/gram tissue. Similar to the bacterial inoculums, no significant differences were identified. Since the CFU counts are corrected for tissue sample weight, this does not represent the absolute total CFU count post-termination. Please note that all tissues of murine hind legs injected with Cytodex beads were culture-negative post-termination. This rules out a possible cross-contamination of the beads through blood-borne dissemination of bacteria from the contralateral infected leg.

Table S2: Tracer biodistribution

	[18F]BODIPY-FL-vancomycin	[18F]PQ-VE1-vancomycin	[18F]FDG
Organ	%ID/g ± SD	%ID/g ± SD	%ID/g ± SD
Whole blood	4.74 ± 1.37	6.23 ± 1.87	1.50 ± 1.39
Plasma	8.03 ± 2.05	9.55 ± 1.62	1.52 ± 1.14
Heart	3.61 ± 0.98	7.74 ± 1.15	95.28 ± 16.30
Lungs	11.11 ± 2.24	10.53 ± 1.78	12.57 ± 3.36
Liver Lobe	5.32 ± 4.14	12.52 ± 4.45	3.73 ± 1.76
Spleen	5.08 ± 1.07	16.45 ± 3.49	11.79 ± 2.88
Kidney	11.35 ± 6.07	10.71 ± 2.73	6.70 ± 1.73
Bladder	19.19 ± 12.58	10.25 ± 1.10	9.87 ± 4.31
Stomach	4.30 ± 1.15	7.68 ± 1.00	7.28 ± 2.71
Urine	138.8 ± 126.0	13.1 ± 6.3	69.6 ± 79.8
Small Intestine	3.78 ± 1.25	6.88 ± 1.20	6.52 ± 2.10
Large Intestine	3.05 ± 1.34	6.60 ± 2.91	14.02 ± 7.75
Fat	1.28 ± 0.44	1.48 ± 0.34	0.92 ± 0.52
Bone	5.81 ± 1.17	3.79 ± 0.93	9.04 ± 2.40
Brain	0.17 ± 0.04	0.27 ± 0.07	5.19 ± 1.72
Muscle	1.19 ± 0.30	2.41 ± 0.69	2.12 ± 1.04
Leg Left *S. aureus*	3.50 ± 0.50	3.57 ± 0.64	4.47 ± 0.46
Leg Left *E. coli*	3.18 ± 0.70	2.83 ± 0.48	3.98 ± 0.76
Leg Right Cytodex	2.27 ± 0.40	2.96 ± 0.52	2.88 ± 0.45
Leg Right PBS	2.43 ± 0.48	2.80 ± 0.57	2.63 ± 0.69

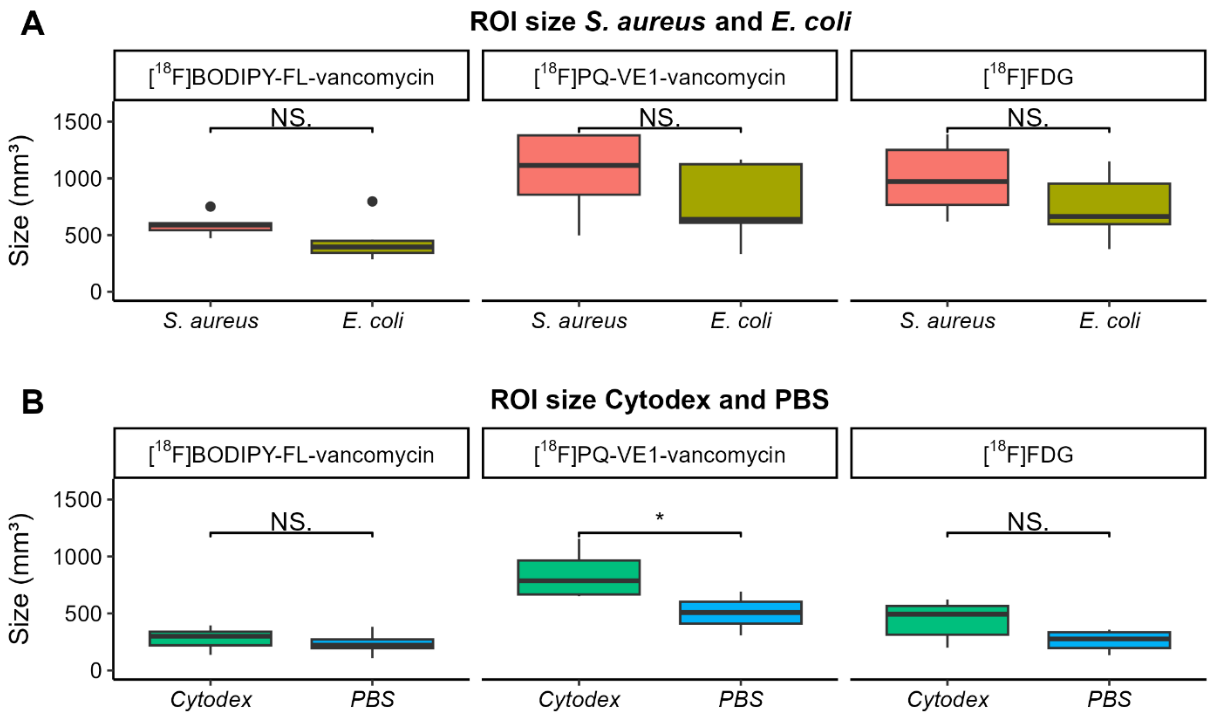

Figure S2: Sizes of regions of interest (ROIs). Data is represented as the median with IQR. Statistical analysis was performed with the Wilcoxon signed rank test and p-values are indicated. In the PET images, ROIs were drawn based on the presence of activity in the respective hindlegs. When looking at the size of the ROIs, there was no significant difference in accumulation size between *S. aureus* and *E. coli* **(A)**, or Cytodex and PBS **(B)**. This, in combination with the SUV_{mean} values from the PET data, implies that the accumulated signal in *S. aureus* infection was not exclusively the result of blood pooling. If that had been the case, no difference would have been observed in the ROI sizes and the SUV_{mean} values. As there are differences in SUV_{mean}, this implies specific binding of ^{18}F-vancomycin at the site of infection.

In vitro experiments

To verify binding of [^{18}F]BODIPY-FL-vancomycin and [^{18}F]PQ-VE1-vancomycin to cellular components of (heat-killed) *E. coli* ATCC 25922, bacteria from planktonic culture or from sonicated biofilms were used. For control, *S. aureus* NCTC 8325 was used.

To analyse tracer binding to planktonic bacteria and heat-killed bacteria, bacteria from frozen -80°C stocks were plated, and single colonies were used to inoculate overnight cultures that were in turn used to inoculate a 30 mL fresh culture in TSB, which was grown to an OD_{600} of 0.2. From this culture, a 20 mL aliquot was centrifuged for 3 min at 2500 relative centrifugal force (rcf). The supernatant was removed, the bacterial pellet was resuspended in 20 mL PBS + 1% BSA, and aliquots of 1 mL were transferred to Eppendorf tubes. From the remaining culture, 1 mL aliquots were transferred to Eppendorf tubes *in triplicate*, which were incubated for 30 min in a water bath set to 99°C to heat-kill the bacteria. After heat-killing, the Eppendorf tubes were centrifuged (13.200 rpm, 1 min), supernatant was removed, and the pellet was resuspended in 1 mL PBS + 1% BSA. For CFU-counting of the planktonic bacteria and the biofilm sonicates, 20 µL aliquots from the fresh culture or two biofilm sonicates per bacterial strain were used as above.

To grow biofilms, bacteria from a -80°C stock were plated and incubated for 24 h at 37°C. Single colonies were picked to inoculate 4-mL TSB overnight cultures. Next day, TSB supplemented with 2.5% glucose and 2.5% NaCl was inoculated with an aliquot of the overnight culture to an OD_{600} of 0.05. The inoculated TSB was then transferred to a large Petri dish with 13 mm glass coverslips (Thermo Fisher, Waltham, USA) such that the coverslips were fully submerged. After 24 h of non-shaking incubation at 37°C, medium was refreshed, and incubation was continued for another 24 h. After transfer of individual coverslips to 50 mL Falcon tubes with 10 mL PBS and 1% bovine serum albumin (BSA), bacteria were collected from the coverslips by placing the tubes in a sonicator bath (Misonix, Farmingdale, USA) and sonication for 5 cycles of 5 s at an amplitude of 5%. The coverslip was then removed from the sonicate, which was subsequently centrifuged for 3 min at 3000 rcf (Allegra X-12R, Beckman Coulter, Pasadena, USA) to collect bacteria released from the biofilm. The supernatant of the sonicate was removed, and the bacterial pellet was resuspended in 1 mL PBS + 1% BSA and transferred to a 1.5 mL Eppendorf tube.

Lastly, all bacterial samples were incubated with 150 kBq in 12.5 µL of tracer (< 10% EtOH, 42.8 pg and 0.6 pg for [^{18}F]BODIPY-FL-vancomycin and [^{18}F]PQ-VE1-vancomycin, respectively) for 20 min at room temperature. The samples were then centrifuged (8000 rpm, 1 min) and washed twice with PBS + BSA 1%. Tracer activity in the bacterial pellets was measured using a gamma counter. All experiments were performed *in triplicate*.

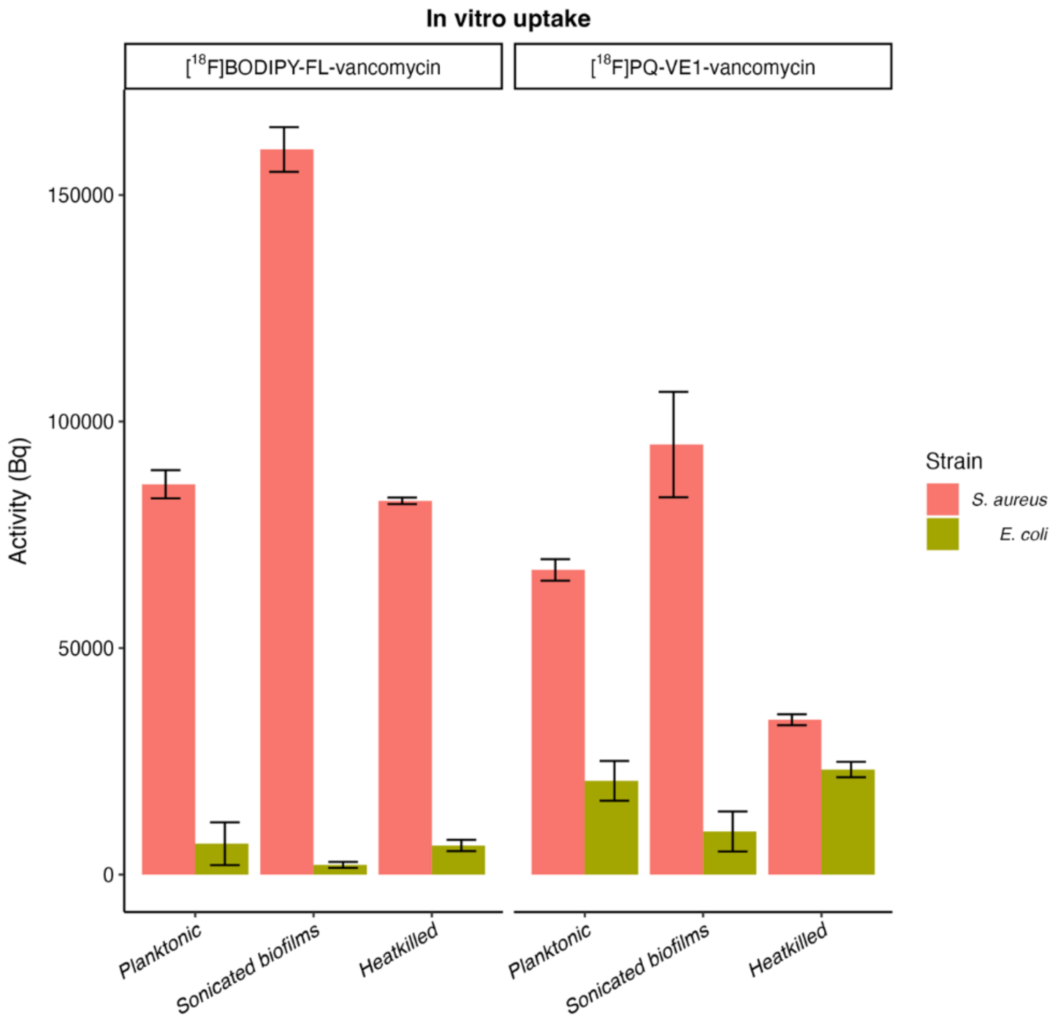

Figure S3: *In vitro* binding of ^{18}F-vancomycin-PET tracers. Data was expressed as the mean ± SD. *In vitro* experiments using planktonic bacteria, bacteria from sonicated biofilms, and heat-killed planktonic bacteria show binding of both ^{18}F-vancomycin-PET tracers to the Gram-positive bacterium *S. aureus*, but not the Gram-negative bacterium *E. coli*. Tracer accumulation was quantified using a calibrated gamma counter after washing the bacterial pellet twice with sterile PBS. Accumulated signal is highest for sonicated biofilms, followed by planktonic and heat-killed bacteria. However, a direct comparison between planktonic and sonicated conditions is challenging as the data could not be corrected for CFUs.
